# Left Atrial Myxoma Hypervascularized from the Right Coronary Artery: An Interesting Cath Lab Finding

**DOI:** 10.1155/2016/4865439

**Published:** 2016-01-12

**Authors:** Marcos Danillo Peixoto Oliveira, Adriano Ossuna Tamazato, Fernando Roberto de Fazzio, Luiz J. Kajita, Expedito E. Ribeiro, Pedro Alves Lemos

**Affiliations:** Department of Interventional Cardiology, Heart Institute (InCor), University of São Paulo, Avenida Dr. Enéas de Carvalho Aguiar 44, 05403-900 São Paulo, SP, Brazil

## Abstract

Primary cardiac tumors are rare and approximately half of them are atrial myxomas. They rarely remain asymptomatic, especially if large. The imaging of a myxoma by contrast dye during coronary angiography is an infrequent sign, which clarifies the vascular supply of the tumor. We report herein an interesting and rare case of a left atrial myxoma hypervascularized from the right coronary artery.

## 1. Introduction

Myxomas are benign and the most common cardiac tumors. They are predominantly located in the left atrium. The clinical presentation varies according to their localization and size. The imaging of such a tumor by contrast media during coronary angiography is a rare finding, which displays the vascular supply of the tumor [1, 2]. We report herein the case of a 39-year-old woman presenting with exertional chest pain due to a left atrial myxoma hypervascularized from the right coronary artery (RCA).

## 2. Case Report

A 39-year-old woman, active, presented with a recent four-month history of exertional chest pain. There were no previous episodes of myocardial infarction, stroke, or coronary artery disease or personal or familiar histories of sudden cardiac death.

**Figure 1:**
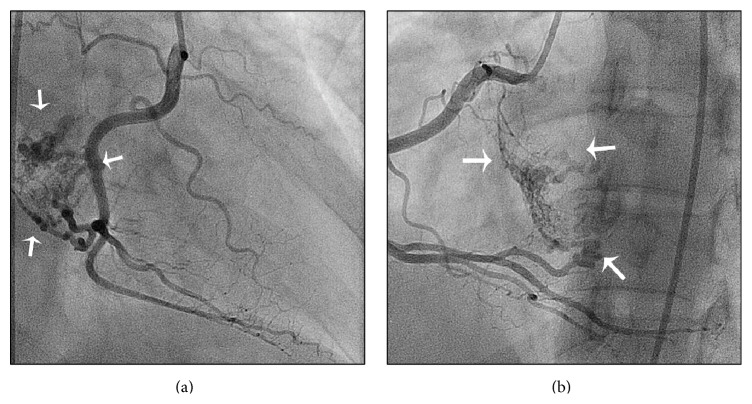
Selective right coronarography showing the large amount of contrast media enhancing the tumoral mass (white arrows). (a) Right anterior oblique projection; (b) left anterior oblique projection.

**Figure 2:**
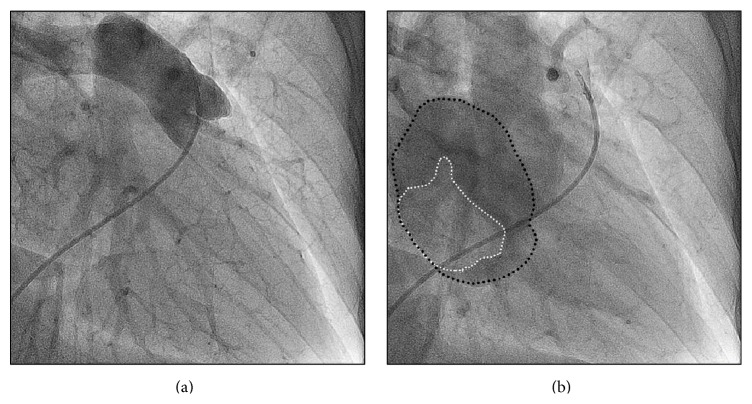
(a) Pulmonary angiography. (b) During the late left atrial filling (black dotted line) by the dye contrast, the negative image corresponding to the tumoral mass presence was clearly noted (white dotted line).

General physical evaluation showed no significant findings. The chest radiography, the resting electrocardiogram, and blood tests showed no relevant alterations. Transthoracic and transesophageal echocardiography revealed a 27 × 38 mm sessile echodense mass attached to the left side of the interatrial septum with mixed hyperechogenic images suggesting a necrotic myxomatous tumor. Before the proposed corrective surgery, a coronary angiography was performed in order to rule out subclinic coronary artery disease. There were no lesions at all. During the selective right coronarography, a large amount of contrast media enhanced the tumoral mass (Figure 1 and video 1 in Supplementary Material available online at http://dx.doi.org/10.1155/2016/4865439). Following the pulmonary angiography, during the late left atrial filling by the dye contrast, the negative image corresponding to the tumoral mass presence was clearly noted (Figure 2 and video 2). During the surgical procedure, the communicating branches of the RCA were detected and ligated. The tumoral mass showed a regular and smooth surface. Its histopathologic examination showed myxoid degeneration, without calcification, with the typical clustered collections of the myxoma cells and intense neovascular structures. The patient was discharged home after three days of the surgery, which was completed uneventfully. She coursed, however, with postpericardiotomy syndrome, which was managed appropriately. At the time of this report, two years after the surgical procedure, the patient is asymptomatic, without new adverse events.

## 3. Discussion

Myxomas are the most frequent benign tumors of the heart. Approximately 85% of them are located in the left atrium [3]. Their clinical manifestations vary according to their anatomic position and size. There are mainly 3 types of presentations: constitutional, embolic, and obstructive. Constitutional symptoms include fever, malaise, loss of appetite, and weight loss. Embolic manifestations include stroke, myocardial infarction, and visceral infarctions. Obstructive manifestations are usually mistaken as mitral or tricuspid valvar stenosis [2].

Beyond ruling out coronary lesions as preoperative assessment, coronary angiography can be useful in diagnosing and evaluating the vascularity of atrial myxomas [1]. In the majority of cases the source of vascularization is the left circumflex artery [4]. Like in our case, only few patients with left atrial myxoma supplied from the RCA have been reported [1, 2, 4].

Angiographic visualization of the feeding vessels has several clinical and therapeutic implications. The detection of these vessels can influence the surgical strategy in cases with evidence of blood shunting, due to either spurting from the myxoma surface or fistula formation. This can result in a steal phenomenon that will lead many surgeons to ligate these feeding vessels during the surgical procedure [4, 5].

Coronary angiography can be valuable in differentiating cardiac myxoma from thrombi, which have different therapeutic approaches (surgery and anticoagulation, resp.). The presence of neovascularization favors the diagnosis of a cardiac myxoma rather than thrombus, which is most often nonvascularized [4].

Surgical excision is the definitive treatment and should not be delayed especially with polypoid types because of the high incidence of embolization. Adequate excision of the entire mass prevents recurrence. Regular follow-up by noninvasive methods is mandatory for early detection of tumoral recurrence. The mid-term survival is similar to that of the age- and sex-matched population [4].

## Supplementary Material

Video 1: Right coronarography showing the large amount of contrast media enhancing the tumoral mass.
Video 2: Pulmonary angiography. During the late left atrial filling by the dye contrast, the negative image corresponding to the tumoral mass presence was clearly noted.
